# Association between the C-reactive protein-albumin-lymphocyte index and all-cause mortality in Chinese older adults: a national cohort study based on CLHLS from 2014 to 2018

**DOI:** 10.3389/fnut.2025.1575470

**Published:** 2025-05-15

**Authors:** Tian Hu, Taotao Wang, Xiaojing Luo, Zheng Hu

**Affiliations:** ^1^Department of Rehabilitation Medicine, JieYang People’s Hospital, Jieyang, Guangdong, China; ^2^Department of Rehabilitation Medicine, Zhongshan People’s Hospital, Zhongshan, Guangdong, China; ^3^Department of Rehabilitation Medicine, Affiliated Hospital of Guangdong Medical University, Zhanjiang, Guangdong, China; ^4^Department of Spine Surgery, JieYang People’s Hospital, Jieyang, Guangdong, China

**Keywords:** Chinese older people, cohort study, mortality, C-reactive protein-albumin-lymphocyte, Chinese longitudinal healthy longevity survey

## Abstract

**Background:**

The C-reactive protein-albumin-lymphocyte (CALLY) index, a novel inflammation-immune-nutritional biomarker, has not been comprehensively evaluated for mortality risk prediction in older populations. Here, we investigate the relationship between the CALLY index and all-cause mortality in Chinese adults aged ≥ 60 years.

**Methods:**

Data were obtained from the 2014 to 2018 wave of the Chinese Longitudinal Healthy Longevity Survey (CLHLS). Upon applying a natural logarithmic transformation to the CALLY index, the lnCALLY was stratified into tertiles. Kaplan-Meier analysis and the log-rank test were employed to assess the cumulative survival probability across lnCALLY-stratified older adults populations. Cox proportional hazards regression was utilized to investigate the association between lnCALLY and all-cause mortality. Receiver operating characteristic (ROC) curves and area under the curve (AUC) values were conducted to evaluate the predictive capacity of lnCALLY for all-cause mortality. Restricted cubic splines (RCS) with four knots were applied to explore the potential non-linear dose-response association of lnCALLY with all-cause mortality. Subgroup analyses and sensitivity analyses were conducted to ensure validity.

**Results:**

A total of 1,738 older adults participants were included in this cohort. Over a median follow-up of 3.3 years, 580 deaths (33.3%) occurred. The multivariable Cox regression demonstrated that the highest lnCALLY tertile was associated with a 40% reduced mortality risk compared to the lowest tertile [adjusted hazard ratio (HR) = 0.60, 95% confidence interval (CI): 0.49–0.73]. Kaplan-Meier curves revealed significantly higher survival probabilities in individuals with elevated lnCALLY (*P* < 0.001). Time-dependent ROC analysis showed that the AUC of lnCALLY for predicting all-cause mortality at 1-, 2-, and 3-year were 0.751, 0.746, and 0.762, respectively. RCS demonstrated an approximate “L”-shaped negative correlation between lnCALLY and all-cause mortality (*P*_*overall*_ < 0.001, *P*_*non*–_*_*linearity*_* = 0.364). Subgroup and sensitivity analyses confirmed robustness, with no significant interactions observed across demographic or clinical strata.

**Conclusion:**

These findings suggest that the CALLY index serves as a practical prognostic biomarker for monitoring survival in older populations, underscoring the interplay of inflammation, immunity, and nutrition in aging-related mortality.

## 1 Introduction

In China, the aging population is expanding at an unprecedented rate. According to the seventh National Census conducted in 2020, individuals aged 65 and older reached 190 million, accounting for 13.5% of the total population-significantly higher than the global average of 9.3% (1). Projections indicate that this demographic will more than double by 2050, reaching 366 million and comprising 26.0% of the population (2). While this shift reflects improved life expectancy and societal advancement, it also poses critical challenges to sustaining the health and quality of life of the older adults.

Chronic inflammation, malnutrition, and immune dysregulation constitute central mechanisms driving human aging pathogenesis and progression (3–5). Hematological indicators are commonly used in clinical and preclinical studies to assess inflammation, nutrition, and immunity. C-reactive protein (CRP) serves as a highly sensitive inflammatory biomarker reflecting acute inflammatory conditions, such as trauma, infection, and infarction (6), while also providing valuable prognostic information (7–9). Serum albumin, the most abundant circulating protein in the blood, represents a vital nutritional indicator (10). Clinically low serum albumin levels demonstrates strong associations with increased incidence of age-related diseases (11, 12), and serves as an independent risk factor for mortality (13). Lymphocytes, fundamental to immune function, exhibit age-dependent behavior alterations that contribute to immunosenescence, increasing susceptibility to age-related diseases (14). Growing evidence shows that chronic inflammation, malnutrition, and immune dysregulation are not isolated but rather form an interconnected pathological network that collectively drives aging processes (15). Therefore, a single biomarker may not provide sufficient information to assess the health status of the older adults, making the exploration of a comprehensive index based on multiple indicators a promising research direction.

The CRP-albumin-lymphocyte (CALLY) index, an innovative inflammation-immune-nutritional scoring system derived from CRP, serum albumin, and lymphocyte count, has recently received significant attention. First introduced by Lida to predict post-hepatectomy prognosis in hepatocellular carcinoma patients (16), the CALLY index has shown promising applications in prognostic assessments of other malignancies, including non-small cell lung cancer, esophageal cancer, and breast cancer (17–19). Besides, the CALLY index has also been employed to monitor health trajectories in older adults. For example, Li et al. (20) identified low CALLY index levels as a significant risk factor for sarcopenia among the older adults. Similarly, Luo et al. (21) reported a strong association between the CALLY index and risks of all-cause mortality and cardiovascular diseases (CVDs) mortality in aging Americans. Despite these advancements, the predictive role of the CALLY index in the Chinese older adults population remains unclear, particularly given potential regional and ethnic variations. Investigating this association in Chinese older adults carries substantial public health implications, as it provides valuable insights into how controlling inflammation, supporting nutrition, and enhancing immunity to achieve “healthy aging” in this population. We hypothesized that the CALLY index may serve as a strong predictor of all-cause mortality. Therefore, we performed this study based on the 2014-2018 wave of the Chinese Longitudinal Healthy Longevity Survey (CLHLS) to investigate the relationship between the CALLY index and all-cause mortality in Chinese older adults.

## 2 Materials and methods

### 2.1 Data and population source

The longitudinal data for this study were sourced from the CLHLS, a nationally representative, prospective cohort study of older adults initiated by Peking University to investigate social, behavioral, environmental, and biological determinants of health and longevity. Since its initiation in 1998, the CLHLS has conducted follow-up surveys every 3-4 years intervals across 23 Chinese provinces. Detailed information regarding the sampling design and data quality assessments of the CLHLS has been thoroughly documented in previous publications (22).

In the present study, we focused on a longitudinal sample of the CLHLS in the 2014 wave. The initial sample consisted of 2,546 participants, with follow-up assessments conducted in 2018. To ensure data integrity, exclusion criteria were applied as follows: (1) absence of biomarker data at baseline (*n* = 69); (2) younger ages (<60 years, *n* = 24); (3) incomplete covariate information (*n* = 492); and (4) absence of follow-up data (*n* = 223). After these exclusions, 1,738 older adults remained in the final sample for analysis. The study received ethical approval from the Research Ethics Committee of Peking University (IRB00001052-13074). All senior participants or their guardians were informed about the survey content and provided their informed consent.

### 2.2 Measurement

#### 2.2.1 Assessment of CALLY

During the baseline survey in 2014, experienced medical staff collected 5 ml of fasting venous blood from older adults participants using heparin-anticoagulated vacuum blood collection tubes. The heparinized blood samples were centrifuged at 2,500 rpm for 10 min to separate the plasma, which was stored at –20°C and sent to the Clinical Laboratory Center of Capital Medical University for analysis of biomedical indicators (23). Plasma albumin and CRP levels were measured in a sequential automated analyzer (Hitachi 7108, Tokyo, Japan). Plasma albumin was measured by bromocresol green assay, while CRP was measured by immunoturbidimetry assay (Roche Diagnostic, Mannheim, Germany) (24, 25). Routine blood tests were performed to measure red blood cell count, white blood cell count, platelet count, and hemoglobin levels. According to previous studies (21, 26), the CALLY index was calculated using the formula:


CALLY⁢index

$$=\frac{\mathrm{Albumin}\,\left(g/\mathrm{dL}\right)\times \mathrm{Absolute}\,\mathrm{Lymphocyte}\,\mathrm{Count}\,\left(\mathrm{cells}/\mathrm{uL}\right)}{\mathrm{CRP}\,\left(\mathrm{mg}/\mathrm{dL}\right)\times 10^{4}}$$

As the CALLY index was not normally distributed, a natural logarithm (ln) transformation was applied prior to subsequent analyses. Participants were stratified into tertiles based on lnCALLY: Tertile 1 (lnCALLY ≤ 1.42), Tertile 2 (1.42 < lnCALLY < 2.47), and Tertile 3 (lnCALLY ≥ 2.47).

#### 2.2.2 Assessment of mortality

In the follow-up surveys in 2018, the survival status of older individuals was confirmed. For deceased individuals, mortality date and related information were collected through questionnaires and interviews from their family members. Survival time was defined as the interval between the baseline survey date and mortality date. A “loss to follow-up” was defined as the inability to contact the participants or their family members in the 2018 follow-up waves. For survivors, survival time was calculated as the interval between the 2018 follow-up date and the baseline survey date.

#### 2.2.3 Assessment of covariates

Covariates were collected through a structured questionnaire at baseline, encompassing demographic characteristics, such as age, sex, place of residence, marital status, educational background, body mass index (BMI), smoking status and drinking status, as well as chronic diseases, including hypertension, diabetes, CVDs, and stroke. BMI was calculated as body weight (kg) divided by height squared (m^2^). Hypertension was defined as systolic blood pressure ≥ 140 mmHg, diastolic blood pressure ≥ 90 mmHg, or self-reported physician diagnosis. Diabetes was diagnosed through fasting blood glucose level ≥ 7.0 mmol/L or self-reported physician confirmation. Additionally, the history of CVDs and stroke was all self-reported.

### 2.3 Statistical analysis

Quantile-quantile (Q-Q) plots were used to assess the normality of continuous variables. Continuous variables were presented as mean (standard deviation) for normally distributed data or median (interquartile ranges) for non-normally distributed data. Categorical variables were expressed as number (frequency). Characteristics among the three lnCALLY groups were compared using One-way ANOVA test (normally distributed continuous variables), Kruskal-Wallis *H*-test (non-normally distributed continuous variables), or Chi-squared tests (categorical variables).

Kaplan-Meier analysis with log-rank test was employed to assess the cumulative survival probability across older adults groups stratified by lnCALLY tertiles. The Cox proportional hazards models were used to investigate the association between lnCALLY index and all-cause mortality risk. Hazard ratios (HRs) with corresponding 95% confidence intervals (CIs) were reported for three progressively adjusted models: Model 1 was unadjusted; Model 2 was adjusted for demographic characteristics (age, sex, place of residence, marital status, educational background, BMI, smoking status and drinking status); and Model 3 further adjusted for chronic diseases (hypertension, diabetes, CVDs, and stroke) based on Model 2. The predictive capacity of the lnCALLY for all-cause mortality was evaluated using receiver operating characteristic (ROC) curves for three models, supplemented by time-dependent ROC analysis for Model 3. Corresponding area under the curve (AUC) values were quantified to assess discrimination accuracy.

The restricted cubic splines (RCS) with four knots at the 25th, 50th, 75th, and 95th centiles were applied to further explore the association of lnCALLY with all-cause mortality. The potential for a nonlinear dose-response relationship was evaluated using the likelihood ratio test.

To assess the robustness of the findings, subgroup and sensitivity analyses were conducted. Subgroup analyses evaluated potential heterogeneity in associations across strata defined by age, sex, place of residence, and BMI. Interaction *p*-values were derived from likelihood ratio tests comparing regression models with and without interaction terms. Sensitivity analyses included: (1) exclusion of participants aged < 65 years or > 105 years; (2) removal of individuals who died within the first year of follow-up; and (3) multiple imputation with chained equations to address missing covariate data.

All statistical analyses were conducted using R software, version 4.1.3, and IBM SPSS, version 26.0. A two-sided *P* < 0.05 was considered statistically significant.

## 3 Results

### 3.1 Baseline characteristics

Among 1,738 Chinese older adults participants, approximately 62.6% (*n* = 1,088) were aged > 80 years, 49.65% (*n* = 863) were males, and the majority (80.26%) resided in rural. The baseline characteristics of participants, stratified by tertiles of lnCALLY, were shown in Table 1. Compared with the lowest tertile, participants in the highest lnCALLY tertile exhibited significantly higher proportions of individuals aged ≤ 80 years, married individuals, literate individuals, and non-overweight individuals (all *P* < 0.05). Additionally, a lower prevalence of diabetes history was also observed in the highest tertile (*P* < 0.05).

**TABLE 1:** The baseline characteristics of the Chinese older participants.

Characteristics	Total(*n* = 1,738)	Tertiles of lnCALLY			*P*-value
		**Tertile 1**	**Tertile 2**	**Tertile 3**	
Age (years), n (%)					<0.001
≤80	650 (37.40)	161 (27.81)	230 (39.72)	259 (44.66)	
>80	1088 (62.60)	418 (72.19)	349 (60.28)	321 (55.34)	
Sex, n (%)					0.297
Male	863 (49.65)	287 (49.57)	301 (51.99)	275 (47.41)	
Female	875 (50.35)	292 (50.43)	278 (48.01)	305 (52.59)	
Place of residence, n (%)					0.246
Urban	343 (19.74)	117 (20.21)	124 (21.42)	102 (17.59)	
Rural	1395 (80.26)	462 (79.79)	455 (78.58)	478 (82.41)	
Marital status, n (%)					<0.001
Married	722 (41.54)	197 (34.02)	239 (41.28)	286 (49.31)	
Single	1016 (58.46)	382 (65.98)	340 (58.72)	294 (50.69)	
Educational background, n (%)					0.034
Illiteracy	1054 (60.64)	375 (64.77)	333 (57.51)	346 (59.66)	
Literacy	684 (39.36)	204 (35.23)	246 (42.49)	234 (40.34)	
BMI (kg/m^2^), n (%)					0.026
<18.5	316 (18.18)	117 (20.21)	82 (14.16)	117 (20.17)	
18.5-23.9	982 (56.50)	322 (55.61)	333 (57.51)	327 (56.38)	
≥ 24	440 (25.32)	140 (24.18)	164 (28.32)	136 (23.45)	
Smoking status, n (%)					0.460
Never	1323 (76.12)	440 (75.99)	447 (77.20)	436 (75.17)	
Past	138 (7.94)	54 (9.33)	40 (6.91)	44 (7.59)	
Current	277 (15.94)	85 (14.68)	92 (15.89)	100 (17.24)	
Drinking status, n (%)					0.228
Never	1375 (79.11)	454 (78.41)	462 (79.79)	459 (79.14)	
Past	91 (5.24)	40 (6.91)	27 (4.66)	24 (4.14)	
Current	272 (15.65)	85 (14.68)	90 (15.54)	97 (16.72)	
Hypertension, n (%)					0.487
Yes	1109 (63.81)	377 (65.11)	373 (64.42)	359 (61.90)	
No	629 (36.19)	202 (34.89)	206 (35.58)	221 (38.10)	
Diabetes, n (%)					0.005
Yes	208 (11.97)	90 (15.54)	59 (10.19)	59 (10.17)	
No	1530 (88.03)	489 (84.46)	520 (89.81)	521 (89.83)	
CVDs, n (%)					0.417
Yes	165 (9.49)	54 (9.33)	62 (10.71)	49 (8.45)	
No	1573 (90.51)	525 (90.67)	517 (89.29)	531 (91.55)	
Stroke, n (%)					0.422
Yes	105 (6.04)	37 (6.39)	39 (6.74)	29 (5.00)	
No	1633 (93.96)	525 (90.67)	517 (89.29)	531 (91.55)	
CRP (mg/dL), M(IQR)	0.11 [0.05, 0.25]	0.36 [0.23, 0.69]	0.12 [0.09, 0.15]	0.04 [0.03, 0.06]	<0.001
Albumin (g/dL), M(IQR)	4.32 [4.09, 4.52]	4.17 [3.92, 4.39]	4.36 [4.12, 4.54]	4.39 [4.22, 4.58]	<0.001
Lymphocytes (1,000 cells/μL), M(IQR)	1.82 [1.41, 2.37]	1.60 [1.20, 2.10]	1.90 [1.50, 2.39]	2.06 [1.64, 2.58]	<0.001

CALLY, C-reactive protein-albumin-lymphocyte index; BMI, body mass index; CVDs, cardiovascular diseases; CRP, C-reactive protein.

### 3.2 Associations of lnCALLY and all-cause mortality

**FIGURE 1:**
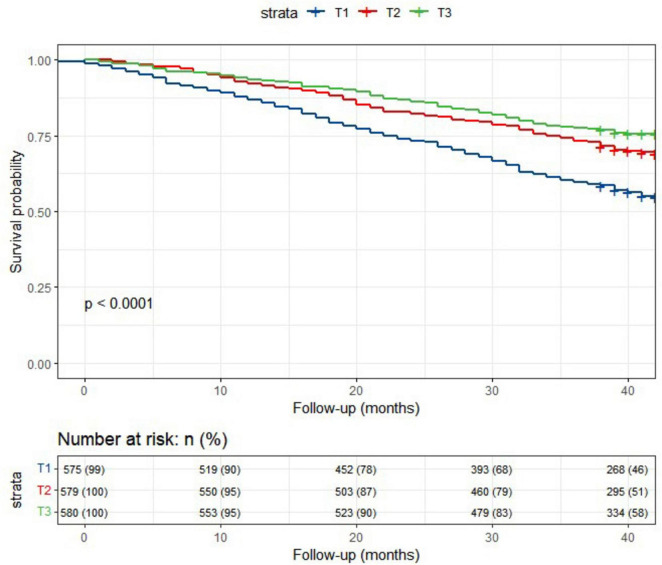
Kaplan-Meier survival curves depicted the survival rate and the number (%) of at-risk Chinese older adults individuals for all-cause mortality, stratified by lnCALLY tertiles.

In the current study, a total of 5040.2 person-years of follow-up were accumulated. Over a median follow-up period of 3.3 years, 580 deaths (33.3%) were recorded. Kaplan-Meier survival analysis (Figure 1) revealed that older participants with a higher lnCALLY had significantly greater survival rates compared to those with a lower lnCALLY (*P* < 0.001). Specifically, individuals in the T3 exhibited a lower risk of all-cause mortality compared to those in the other two tertiles.

As shown in Table 2, using the T1 of lnCALLY as the reference, the T3 was inversely associated with the risk of all-cause mortality across all three models (Model 1: HR = 0.48, 95% CI: 0.39-0.58; Model 2: HR = 0.59, 95% CI: 0.48-0.73; Model 3: HR = 0.60, 95% CI: 0.49-0.73). A significant dose-response relationship was observed in all three models (*P* for trend < 0.001). Additionally, each standard deviation (SD) increase in the lnCALLY was significantly associated with reduced mortality risk across all models (Model 1: HR = 0.73, 95% CI: 0.68-0.79; Model 2: HR = 0.81, 95% CI: 0.75-0.88; Model 3: HR = 0.81, 95% CI: 0.75-0.88).

**TABLE 2:** Associations of lnCALLY and all-cause mortality risk in Chinese older participants.

	lnCALLY, HR (95% CI)					
	**Tertile 1**	**Tertile 2**	**Tertile 3**	***P* for trend**	**Per SD increase**	***P*-value**
Model 1^a^	1.00	0.61 (0.50-0.73)	0.48 (0.39-0.58)	<0.001	0.73 (0.68-0.79)	<0.001
Model 2^b^	1.00	0.72 (0.60-0.88)	0.59 (0.48-0.73)	<0.001	0.81 (0.75-0.88)	<0.001
Model 3^c^	1.00	0.72 (0.59-0.87)	0.60 (0.49-0.73)	<0.001	0.81 (0.75-0.88)	<0.001

CALLY, C-reactive protein-albumin-lymphocyte index; HR, hazard ratio; CI, confidence interval.

*^a^*Model 1: did not adjust any covariates.

*^b^*Model 2: adjusted for age, sex, place of residence, marital status, educational background, BMI, marital status, smoking status, drinking status.

*^c^*Model 3: adjusted all covariates.

### 3.3 Predictive capacity of the lnCALLY for all-cause mortality

**FIGURE 2:**
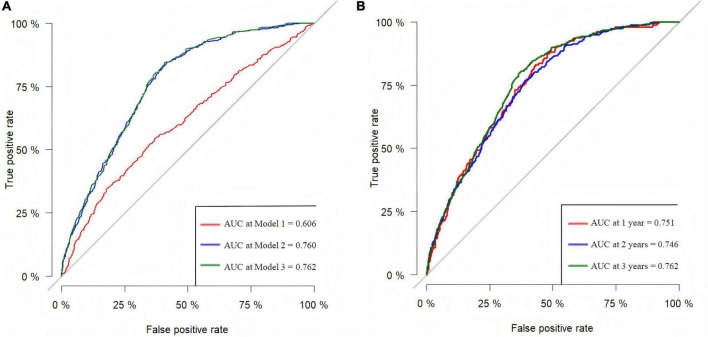
ROC curves for predicting all-cause mortality in the older adults. **(A)** ROC curves for predicting the risk of all-cause mortality for three models. **(B)** Predictive capability of time-dependent ROC assessment of lnCALLY for 1-, 2-, and 3-year all-cause mortality in Model 3.

Figure 2A displayed ROC curves assessing the predictive capacity of Model 1, Model 2, and Model 3 for all-cause mortality in older adults. The AUC values for lnCALLY in three models were 0.606, 0.760, and 0.762, respectively. Figure 2B showed time-dependent ROC curves evaluating the predictive value of predicting all-cause mortality for lnCALLY at 1-, 2-, and 3-year. The corresponding AUC values for lnCALLY at these time points were 0.751, 0.746, and 0.762, respectively.

### 3.4 A non-linear dose-response relationship between lnCALLY and all-cause mortality

**FIGURE 3:**
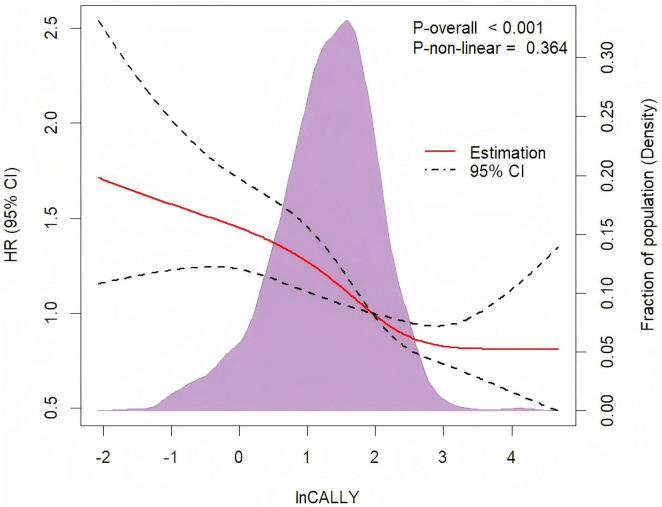
Restricted cubic spline regression with four knots was adjusted for age, sex, place of residence, marital status, educational background, BMI, smoking status, drinking status, hypertension, diabetes, cardiovascular diseases, and stroke to examine the association between lnCALLY and all-cause mortality.

The non-linear dose-response relationship between lnCALLY and all-cause mortality was assessed using a four-knot RCS. As depicted in Figure 3, the multivariate Cox regression model with RCS demonstrated an approximate “L”-shaped negative correlation between lnCALLY and all-cause mortality (*P*_*overall*_ < 0.001, *P*_*non*–_*_*linearity*_* = 0.364). Threshold effect analysis was shown in Table 3.

**TABLE 3:** Non-linearity addressing of lnCALLY and all-cause mortality.

Outcome	HR, 95%CI
Model 1: Fitting model of standard multi-factor COX regression analysis model	0.853 (0.803-0.906)
Model 2: Fitting model of two-piecewise multi-factor COX regression analysis model	
Inflection point	–0.505
<-0.505	1.146 (0.794-1.654)
>-0.505	0.821 (0.761-0.885)
P for log likelihood ratio test	0.092

HR, hazard ratio; CI, confidence interval.

### 3.5 Subgroup analysis

**FIGURE 4:**
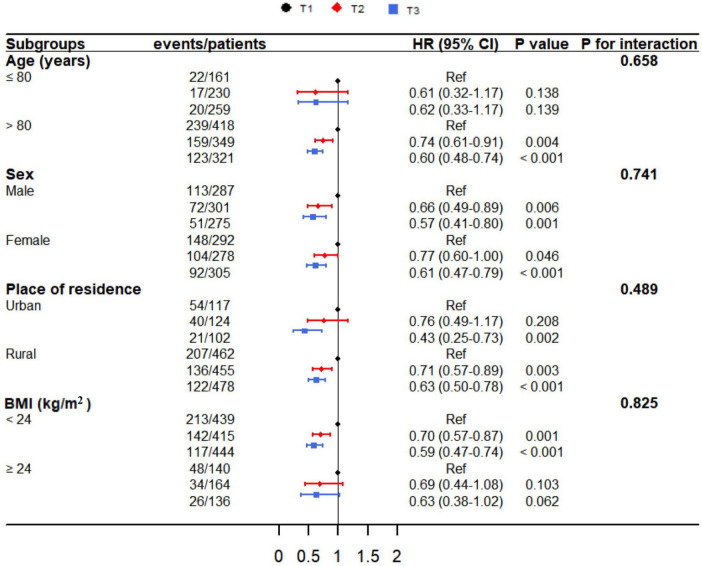
The relationship between lnCALLY and all-cause mortality, stratified by age, sex, place of residence, and BMI.

Figure 4 showed the subgroup analyses stratified by age, sex, place of residence, and BMI. No significant interactions were identified between lnCALLY and prespecified subgroup variables (*P* for interaction > 0.05).

### 3.6 Sensitivity analysis

Sensitivity analyses were conducted to assess the robustness of the primary findings, including: (1) exclusion of participants aged < 65 years or > 105 years; (2) removal of individuals who died within the first year of follow-up; and (3) multiple imputation with chained equations to address missing covariate data (Table 4). These analyses consistently demonstrated an inverse association between lnCALLY and all-cause mortality (*P* < 0.05), confirming the robustness of Cox regression-derived findings.

**TABLE 4:** Sensitivity analysis.

	lnCALLY, HR (95% CI)					
	**Tertile 1**	**Tertile 2**	**Tertile 3**	***P* for trend**	**Per SD increase**	***P*-value**
**(1) Excluding participants aged < 65 years or > 105 years**						
Model 1^a^	1.00	0.63 (0.51-0.76)	0.51 (0.41-0.63)	<0.001	0.75 (0.70-0.81)	<0.001
Model 2^b^	1.00	0.73 (0.60-0.89)	0.62 (0.50-0.76)	<0.001	0.82 (0.76-0.89)	<0.001
Model 3^c^	1.00	0.73 (0.60-0.89)	0.62 (0.50-0.76)	<0.001	0.82 (0.76-0.89)	<0.001
**(2) Excluding participants early deaths ≤ 1-year post-enrollment**						
Model 1^a^	1.00	0.62 (0.49-0.77)	0.48 (0.38-0.61)	<0.001	0.76 (0.69-0.83)	<0.001
Model 2^b^	1.00	0.73 (0.58-0.91)	0.58 (0.45-0.73)	<0.001	0.83 (0.76-0.91)	<0.001
Model 3^c^	1.00	0.73 (0.58-0.91)	0.58 (0.46-0.74)	<0.001	0.83 (0.76-0.91)	<0.001
**(3) Multiple imputation with chained equations to address missing covariate data**						
Model 1^a^	1.00	0.61 (0.51-0.71)	0.48 (0.40-0.57)	<0.001	0.72 (0.68-0.77)	<0.001
Model 2^b^	1.00	0.70 (0.59-0.82)	0.58 (0.49-0.69)	<0.001	0.79 (0.74-0.85)	<0.001
Model 3^c^	1.00	0.70 (0.60-0.83)	0.59 (0.50-0.70)	<0.001	0.79 (0.74-0.85)	<0.001

CALLY, C-reactive protein-albumin-lymphocyte index; HR, hazard ratio; CI, confidence interval.

^a^Model 1: did not adjust any covariates.

^b^Model 2: adjusted for age, sex, place of residence, marital status, educational background, BMI, marital status, smoking status, drinking status.

^c^Model 3: adjusted all covariates.

## 4 Discussion

In this prospective cohort study with a 4-year follow-up, we observed a marked inverse link between lnCALLY and all-cause mortality in the Chinese older adults. This finding highlights the mortality risk posed by low CALLY levels in older adults and reveals the potential of the CALLY index as a strong predictor of long-term mortality.

The CALLY index, which integrates CRP, albumin, and lymphocyte count, offers a multidimensional evaluation of inflammatory, nutritional, and immune status. While initially validated for diagnosing and prognosticating various malignancies (17–19), its applicability may extend to geriatric populations, where chronic low-grade inflammation state (CLIS), malnutrition, and immunosenescence are hallmark features of aging (3–5). These statuses form the theoretical foundation for applying the CALLY index to assess the health of the older adults.

CLIS has been identified as a key driver of the aging process (27), characterized by a persistent, sterile, nonspecific, and systemic mild inflammatory background (28). CLIS contributes to aging through a self-reinforcing positive feedback mechanism. Transcriptome sequencing studies have demonstrated that inflammation-related genes and signaling pathways are over-expressed in aging tissues (29, 30), resulting in elevated plasma levels of pro-inflammatory cytokines such as interleukin-1 (IL-1), interleukin-6 (IL-6), and tumor necrosis factor-α (TNF-α) (15, 31). These mediators can accelerate aging via both autocrine and paracrine mechanisms (32, 33). CRP is a stable biomarker for CLIS monitoring, with elevated concentrations closely linked to an increased risk of age-related diseases (e.g., CVDs, neurodegeneration, and chronic metabolic diseases) and mortality (34). Notably, CRP is an effective biomarker in population health surveillance. Previous studies in Chinese communities have linked elevated CRP levels to an increased risk of all-cause mortality among older adults (35, 36). Our findings supported these theories and research, as deceased participants exhibited significantly higher CRP levels compared to survivors.

Malnutrition affects approximately 25% of the global older adults population (37), with long-term undernutrition detrimental to survival and increasing the risk of all-cause mortality by 2.71 times (38). Serum albumin, a vital nutritional marker, shows a dose-dependent relationship with mortality in older individuals: each 5 g/L reduction correlates with a 25% increase in mortality risk (25). Notably, a bidirectional relationship links malnutrition and CLIS (39). CLIS drives malnutrition through two synergistic mechanisms: (1) overexpression of pro-inflammatory cytokines that dysregulate hypothalamic appetite control (manifesting as the “anorexia of aging” phenotype) (40), and (2) concurrent activation of ubiquitin-proteasome-mediated proteolysis and suppression of anabolic pathways like mTOR signaling, leading to systemic protein depletion (4). Conversely, malnutrition aggravates inflammatory by impairing antioxidant defenses and destabilizing immunometabolic homeostasis, thereby amplifying oxidative stress and sustaining pro-inflammatory cytokine overproduction (39, 41). This synergistic pathology underscores the clinical relevance of integrated biomarkers like the CRP-albumin ratio (CAR), which demonstrates prognostic value for mortality stratification in both critically ill patients (42–44) and community-dwelling older adults (45). These findings validate the integration of inflammatory and nutritional metrics in geriatric risk assessment.

Immunosenescence is prevalent in the older adults, which is featured includes thymic involution, naïve-to-memory cell ratio imbalance, metabolic dysregulation, and epigenetic alterations (46). Our study identified a consistent association between lymphopenia and elevated mortality risk, further emphasizing the pivotal role of robust immune function in preserving health among older adults.

Inflammatory and immunosenescence exhibit bidirectional promotion. CLIS exacerbates immunosenescence by driving immune cell depletion. Elevated systemic levels of pro-inflammatory cytokines, such as IL-1, IL-6, and TNF-α, disrupt lymphocyte proliferation, differentiation, and effector responses, thereby driving progressive immunodeficiency (47). Conversely, age-related immune remodeling profoundly disrupts inflammatory homeostasis. In chronic infections, diminished T-cell-mediated pathogen clearance in older individuals fosters persistent antigen exposure and unresolved inflammation, driving inflammatory signaling and cellular senescence (48). Additionally, the development, maintenance and optional functioning of immune cells is dependent on adequate nutrition (49), particularly dietary proteins and micronutrients. Essential amino acids serve as critical substrates for synthesizing immunological proteins, including cytokines and immunoglobulins that orchestrate adaptive immune responses (50). Micronutrients such as zinc, selenium, and vitamins A/D/C exhibit pleiotropic roles in maintaining immune cell signaling pathways and oxidative homeostasis (49). Notably, older adults populations demonstrate increased vulnerability to nutritional deficiencies through age-related physiological declines, including diminished intestinal absorption efficiency, mastication and dysphagia, and anorexia of aging. Compromised nutritional status exacerbates the natural immunosenescence process, creating a vicious cycle of micronutrient insufficiency and progressive immune dysfunction.

Our study is the first to establish the applicability of the CALLY index for monitoring adverse outcomes in community-dwelling older adults populations in China. However, several limitations must be acknowledged. First, CRP, serum albumin, and lymphocyte count were measured at a single timepoint at baseline, which limits our ability to assess longitudinal fluctuations or establish temporal relationships with mortality. Second, although our multivariate models incorporated key confounders, residual confounding from unmeasured variables (e.g., lifestyle, dietary patterns, and genetic polymorphisms) may persist, potentially influencing the observed association between the CALLY index and mortality. Finally, while the CALLY index shows prognostic promise, its foundational pathophysiological mechanisms require systematic validation through targeted mechanistic studies.

## 5 Conclusion

In conclusion, our study found the significant negative association between the CALLY index and all-cause mortality in Chinese older adults individuals, emphasizing the interaction of inflammation, nutrition, and immunity in their overall health. As an efficient and convenient scoring system, the CALLY index has the potential to serve as a valuable biomarker for monitoring the health of older adults in the community.

## Data Availability

Publicly available datasets were analyzed in this study. This data can be found at: Chinese Longitudinal Healthy Longevity Survey (http://opendata.pku.edu.cn/).
